# Galectin-3 alters the lateral mobility and clustering of β1-integrin receptors

**DOI:** 10.1371/journal.pone.0184378

**Published:** 2017-10-10

**Authors:** Esther H. Yang, Julia Rode, Md. Amran Howlader, Marina Eckermann, Jobette T. Santos, Daniel Hernandez Armada, Ruixiang Zheng, Chunxia Zou, Christopher W. Cairo

**Affiliations:** Alberta Glycomics Centre, Department of Chemistry, University of Alberta, Edmonton Alberta, Canada; University of British Columbia, CANADA

## Abstract

Glycoprotein receptors are influenced by myriad intermolecular interactions at the cell surface. Specific glycan structures may interact with endogenous lectins that enforce or disrupt receptor-receptor interactions. Glycoproteins bound by multivalent lectins may form extended oligomers or lattices, altering the lateral mobility of the receptor and influencing its function through endocytosis or changes in activation. In this study, we have examined the interaction of Galectin-3 (Gal-3), a human lectin, with adhesion receptors. We measured the effect of recombinant Gal-3 added exogenously on the lateral mobility of the α5β1 integrin on HeLa cells. Using single-particle tracking (SPT) we detected increased lateral mobility of the integrin in the presence of Gal-3, while its truncated C-terminal domain (Gal-3C) showed only minor reductions in lateral mobility. Treatment of cells with Gal-3 increased β1-integrin mediated migration with no apparent changes in viability. In contrast, Gal-3C decreased both cell migration and viability. Fluorescence microscopy allowed us to confirm that exogenous Gal-3 resulted in reorganization of the integrin into larger clusters. We used a proteomics analysis to confirm that cells expressed endogenous Gal-3, and found that addition of competitive oligosaccharide ligands for the lectin altered the lateral mobility of the integrin. Together, our results are consistent with a Gal-3–integrin lattice model of binding and confirm that the lateral mobility of integrins is natively regulated, in part, by galectins.

## Introduction

Galectins are a family of animal lectins well known to oligomerize glycoprotein receptors, a feature typically ascribed to their multivalent structure.[1],[2] There are 15 known human galectins, which are classified into three structural families.[3] Galectins are either multivalent or able to oligomerize, and their ligands on the cell surface often contain multiple binding sites. Thus, one of the key functions of galectins is their modulation of cell surface receptor organization. Galectin-ligand interactions are generally thought to form oligomer or lattice structures which may regulate the function of receptors on the cell surface.[4–7] The typical ligand motif for galectins includes a terminal β-galactoside, a binding epitope that can be masked by sialylation of glycans.[8],[9] Galectins are known to regulate a number of pathways including apoptosis,[10] immune tolerance, inflammation,[11] and cell adhesion.[12]

In the case of Galectin-3 (Gal-3; also referred to as Mac-2 or LGALS3),[13] the protein is not a covalent dimer. Oligomerization of Gal-3 is largely mediated by the N-terminal domain, which may involve binding of phospho–Ser and–Thr sites.[14] However, the truncated C-terminal domain can also oligomerize in the presence of ligand,[15] and on the cell surface.[16] The crosslinking of receptors by Gal-3 can result in attenuation and activation of signaling pathways,[17] as well as processes including proliferation,[18] phagocytosis,[19] endocytosis,[20, 21] and atherosclerosis.[22] Importantly, Gal-3 has been implicated in the regulation of cell adhesion.[23] Gal-3 enhances leukocyte adhesion,[24]^,^[25] and metastasis in cancer cells.[26–29]

Galectin-3 may mediate cellular pathways by crosslinking of receptors *in cis*, or through bridging of receptors to extracellular targets *in trans*. The affinity of Gal-3 for oligosaccharides has been studied by calorimetry and mass spectrometry, and its highest affinity ligands tend to be *N*-acetyl-lactosamine (LacNAc) or lacto-*N*-neotetraose (LNnT) analogs.[30]^,^[31] Specific glycoprotein targets of Gal-3 include extracellular matrix proteins, such as laminin and fibronectin (FN).[32] Gal-3 can stabilize focal adhesions,[33] and regulate remodeling of extracellular matrix.[34] Gal-3 itself has been reported to be a substrate of matrix metalloproteases, which may regulate its function.[35] Adhesion receptors bound by Gal-3 include integrins and immune receptors.[21, 36–39] The oligomerization of integrin receptors by Gal-3 has been observed for a variety of receptors on multiple cell types. Gal-3–mediated clustering has been associated with β1 integrin endocytosis,[40] complex formation of the α3β1 integrin,[41] adhesion of α2β1 integrin to collagen,[42] and regulation of the dynamics of α5β1 integrin complexes.[33] Interaction of Gal-3 with integrins involves terminal galactose residues, and is blocked by the presence of sialic acid residues.[8]^,^[43]

Our group has been interested in the regulatory function of neuraminidase enzymes (NEU; also called sialidases) in adhesion. The activity of NEU may enhance the function of galectins by revealing cryptic binding sites for receptor crosslinking.[44] While there are a number of studies that have used fluorescence microscopy to determine gross changes to galectin-induced receptor oligomerization, few have quantified these effects on galectin ligands on cells. Belardi et al. reported experiments with artificial Galectin-1 (Gal-1) ligands on cells, where they observed reduced lateral mobility and cluster formation.[45] Galectin-1 has been reported to reduce the lateral mobility of the NiV-F viral fusion protein.[46] The binding of Gal-1 to H-Ras has been detected using FLIM-FRET methods.[47] These examples examined effects of Gal-1, a homodimeric galectin. The effects of a monomeric galectin, such as Gal-3, have not been examined in the same detail.[48] In general, the ability of Gal-3 to oligomerize receptors on the cell has been assessed using fluorescence microscopy. Examples of Gal-3 receptor clustering as determined by microscopy include mucins,[49]^,^[50] CD71,[39] CD147,[51] the α3β1 integrin,[52] and the αvβ3 integrin.[53] Galectin-3 binds targets *in vitro* through positive cooperativity,[54] and studies of Gal-3 binding to cellular receptors with FRET has confirmed lattice formation.[16]

We considered that there was a need for the use of quantitative measurements of Gal-3–mediated receptor crosslinking, which could be used to investigate the effect of Gal-3 on adhesion receptors. Aggregated receptors within the membrane will have a larger cross-section than individual receptors, and could therefore show reduced lateral mobility.[55] Receptor crosslinking may also result in binding of intracellular or extracellular components which can influence diffusivity,[17, 56] and in turn regulate intracellular signaling. In previous studies of NEU on integrin–mediated adhesion we found that α5β1 integrin was positively regulated by human NEU.[44] Herein, we investigate the ability of Gal-3 to interact with the α5β1 integrin. We used measurement of integrin lateral mobility by single particle tracking (SPT) as our primary tool.[57] Our results confirmed that Gal-3 altered the lateral mobility of the α5β1 integrin. We confirm that changes in lateral mobility manifested as changes to integrin clustering using fluorescence microscopy. Furthermore, we used exogenous high-affinity oligosaccharides to disrupt Gal-3–integrin interactions, which also led to increased integrin lateral mobility.

## Results

### HeLa cells express Gal-1 and Gal-3

We first confirmed that the cell line used for our experiments had native expression of galectins. We selected HeLa cells as they are an adherent line that are known to express Gal-3 and Gal-1.[58] Cells were grown to confluence, harvested, and lysed. The lysate was passed over an LNnT or Lac affinity column prepared using DVS chemistry.[59] Analysis of the eluent by shotgun proteomics methods confirmed the presence of Gal-1 and Gal-3 in HeLa lysate (**Table A in S1 File**). We then sought to explore the role of these natively expressed galectins in regulation of integrin lateral mobility using this cell line.

### Lateral mobility of integrin receptors was altered by galectin ligands

To examine the role of endogenous galectins in regulating integrin mobility, we tested whether a high-affinity oligosaccharide for Gal-3 influenced integrin mobility. Using single-particle tracking, we measured the lateral mobility of the α5β1 integrin on HeLa cells. Due to the distribution of diffusion coefficients over multiple decades, we interpreted the data as a log-normal distribution, and compared conditions based on their logarithmic means (**Table 1**). The affinity of lactose (Lac) and lacto-N-neotetraose (LNnT) for Gal-3 are reported to be 26 and 2 μM, respectively.[60] The LNnT tetrasaccharide has relatively high affinity for Gal-3C (10.8 μM K_d_),[61] and has low-micromolar affinity to Gal-8 and Gal-9. Integrin mobility was unaffected by Lac treatment, confirming that the osmotic differences between Lac and buffer did not have any detectable changes on lateral mobility (see Table C in S1 File). We found that integrin lateral mobility was positively affected by the addition of LNnT, with a significant increase in mobility (1.3 ± 0.1 x 10^−10^ cm^2^ sec^-1^). This observation may be consistent with the disruption of galectin-integrin interactions on the cell surface; however, the role of alternative targets for LNnT cannot be ruled out.

**Table 1:** Lateral mobility of integrins.

Condition	N	mean‡	log mean‡
**Control (PBS)**	618	1.4 ± 0.2	0.4 ± 0.02
**LNnT**	177	2.5 ± 0.4***	0.6 ± 0.05*
			
**Gal-3C 50 μg mL^-1^**	136	1.0 ± 0.2	0.3 ± 0.03
**Gal-3 25 μg mL^-1^**	485	1.0 ± 0.1	0.3 ± 0.01*
**Gal-3 50 μg mL^-1^**	183	1.8 ± 0.2	0.7 ± 0.05****
			
**NanI**	523	1.0 ± 0.1	0.4 ± 0.02
			

‡, Units are [x 10^−10^ cm^2^ sec^-1^] or [x 10^−2^ μm^2^sec^-1^].

Cells were treated for 0.5 hrs under each condition. p values were calculated by comparison of two normal populations of raw or log-transformed data as indicated. Samples were compared to the indicated control for significance using a student’s t-test.

*, p ≤ 0.05

***, p ≤ 0.005

****, p ≤ 0.0001.

The recognition epitope of many galectins can be masked by sialylation. We considered that removal of Sia by neuraminidase treatment could reveal cryptic galectin binding sites, which might then alter integrin-galectin interactions.[8, 62] Cells were treated with a bacterial neuraminidase (NanI) that we previously found can de-sialylate the β1 integrin on HeLa.[44] Measurement of integrin lateral mobility showed no significant change to the median mobility after NanI treatment. However, there is a notable loss of trajectories at higher mobility (>2 x10^-10^ cm^2^sec^-1^) observed in the profile of diffusion coefficients (see Figure B in S1 File). We observed that the β1 chain of the integrin showed a decrease in molecular weight after NanI treatment, supporting a role for changes in the integrin glycan in regulating its mobility (see Figure C in S1 File).

### Lateral mobility of integrin receptors was increased by exogenous galectin-3

**Fig 1:**
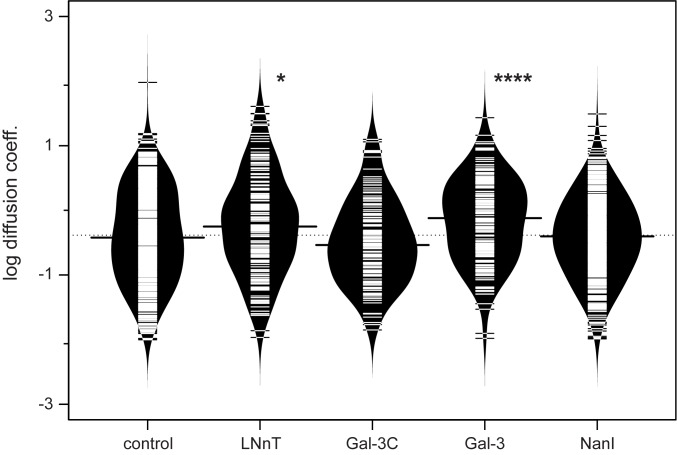
Lateral mobility of integrins is modulated by the presence of saccharides and lectins. The lateral mobility of integrins were determined using SPT, and the data from Table 1 are shown. Each sample population is shown as a bean plot,[87] with the logarithmic median of the diffusion coefficients indicated by a solid line for each population.[87] Each population is shown with a density estimate and horizontal lines indicate individual diffusion coefficient measurements. Gal-3C and Gal-3 treatments are shown for 50 μg mL^-1^ concentrations. Diffusion coefficients are given as log(D), where D is in units of x 10^−10^ [cm^2^s^-1^] or x 10^−2^ [μm^2^s^-1^]. Data were compared to a PBS control using a student’s t-test to determine p values; *, p ≤ 0.05; **, p ≤ 0.01; ***, p ≤ 0.005; ****, p ≤ 0.0001.

Although our results above suggested the involvement of galectins in regulating integrin mobility, other lectin-integrin interactions could be responsible for our observations. To provide more direct evidence of integrin-galectin interactions, we generated recombinant Gal-3 and Gal-3 C-terminal domain (containing only the C-terminal CRD; Gal-3C).[15, 63] The purified proteins were added to HeLa cell culture, and the lateral mobility of the integrin was measured (**Table 1**, **Fig 1**). The addition of Gal-3C at 50 μg mL^-1^ gave a small, but insignificant, reduction in mean lateral mobility of the integrin. The reduction in lateral mobility may be the result of competition for integrin binding sites which do not lead to clustering. There is a notable decrease in the number of trajectories found at higher mobility (>2 x10^-10^ cm^2^sec^-1^) in the Gal-3C profile as compared to the control condition (see Figure B in S1 File). Treatment of cells with Gal-3 showed an opposite effect to that of Gal-3C; with a significant increase in the mean lateral mobility of the integrin when used at 50 μg mL^-1^. The effect of Gal-3 was concentration dependent, as lower concentrations of the Gal-3 protein showed no significant effects (25 μg mL^-1^). The active concentration of Gal-3 is known to vary in different cell types and likely depends on the number of glycan binding sites present and native expression of galectin.[33] These data suggest that a competent Gal-3 protein has opposite effects on lateral mobility relative to the CRD of the lectin alone. In general, trajectories observed for integrins were confined, with very few giving the appearance of free diffusion (c.a. < 10%) as determined by a moment scaling spectrum analysis (MSS).[64]

### Exogenous galectin-3 increased the cluster size of integrins

**Fig 2:**
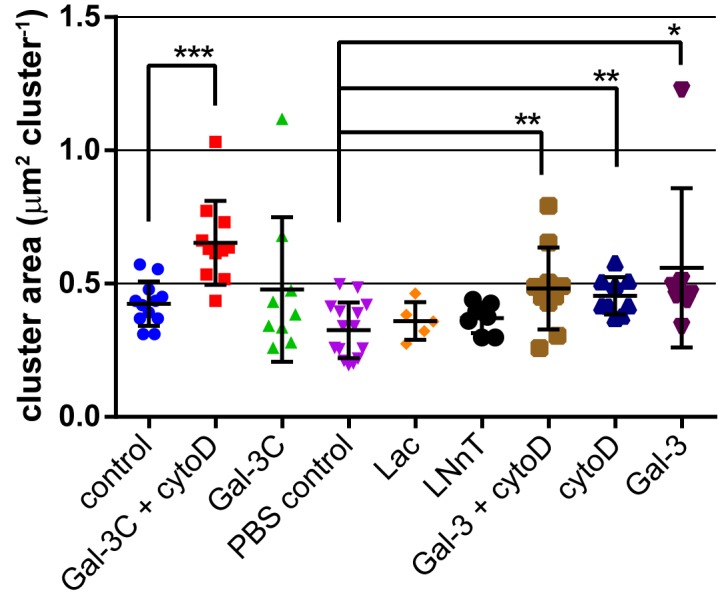
Clustering of integrins is increased on Gal-3 treated cells. Cells were stained using the same anti-α5-Cy5 conjugate employed for tracking experiments. Ten fields of stained cells were analyzed using ImageJ to identify clusters and measure their size. Treatment with Gal-3 resulted in an increase in the size of integrin clusters. See Table B and Figure A in S1 File. Data were compared to a PBS control, or PBS containing BME (control) using a student’s t-test to determine p values; *, p ≤ 0.05; **, p ≤ 0.01; ***, p ≤ 0.005; ****, p ≤ 0.0001.

Although we could observe clear changes in integrin lateral mobility, we also wanted to confirm that these changes altered distribution of the receptor on the cell surface. The distribution of integrin receptors was assessed by acquiring TIRF images of antibody-labelled integrin under each treatment. Images were analyzed for clusters by identifying the pixel area found in clusters on individual cells by thresholding. Treatment of cells with Gal-3C alone did not show a significant increase in integrin cluster size, while treatment with both cytochalasin D (cytoD; a cytoskeletal disruptor) and Gal-3C together increased clustering (**Fig 2**).[65] Cells treated with potential ligands of native galectins, Lac, or LNnT oligosaccharides, did not show any detectable changes in cluster size. Treatment with Gal-3 showed an increase in integrin clustering. Treatment of cells with cytoD alone also resulted in an increase to clustering of the integrin. The effect of Gal-3 and cytoD were not additive, as the combined treatment (Gal-3 + cytoD) gave a similar increase in clustering to each condition alone. We note that as cytoD treatment alone had a similar increase in clustering, the combined treatment does support a substantial effect of Gal-3C on clustering.

### Exogenous galectin-3 increased integrin-mediated cell migration

**Fig 3:**
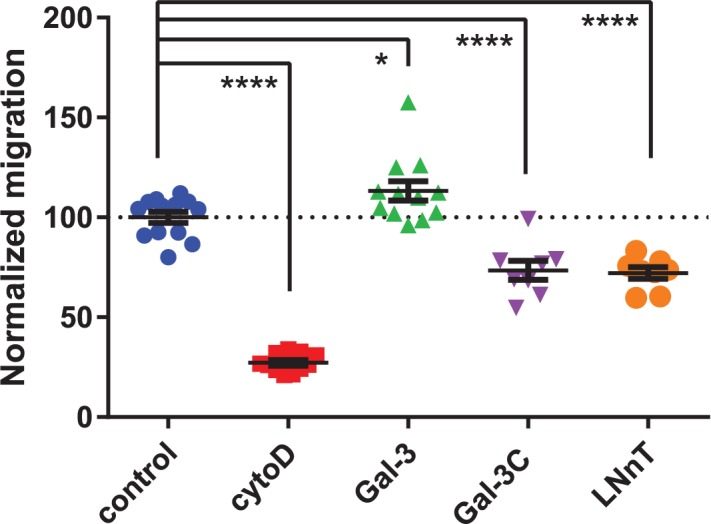
Migration of cells treated with Gal-3, Gal-3C and LNnT. Cells were treated for 21 h with buffer control, Cytochalasin D (197 nM), Gal-3 (50 μg mL^-1^), Gal-3C (50 μg mL^-1^), and LNnT (100 mM). Migration was normalized and compared to buffer control; *, p ≤ 0.05; **, p ≤ 0.01; ***, p ≤ 0.005; ****, p ≤ 0.0001.

To test the functional effect of changes to integrin lateral mobility and cluster size, we examined cell migration in the presence of exogenous galectins (Gal-3 and Gal-3C) and galectin ligands (LNnT). Conditions for migration experiments used the same concentration as those for lateral mobility experiments above. The substrate for cell adhesion was coated with a known β1 integrin ligand, human fibronectin.[66]^,^[67] Cell migration was quantified using a commercial assay to determine net changes in cell coverage over a fixed incubation time (see Materials & Methods). We used cytoD, which depolymerizes actin,[65] as a negative control, which was confirmed to decrease cell migration rates as expected. We found that addition of exogenous Gal-3 caused a moderate increase in cell migration. Interestingly, Gal-3C treatment caused a significant reduction in cell migration. Addition of LNnT also showed a significant decrease in cell migration (**Table 2, Fig 3**). These experiments support that changes observed in lateral mobility and clustering of integrin in the presence of Gal-3 versus Gal-3C could manifest functional changes to integrin activity.

**Table 2:** Normalized β1 integrin-mediated cell migration.

**Condition**	**N**	**migration**†
**Buffer control**	13	100 ± 1
**Cyto D**	11	27 ± 1****
**Gal-3**	12	113 ± 5*
**Gal-3C**	8	74 ± 5****
LNnT	8	72 ± 3****

†Samples were normalized and compared to the indicated control for significance using a Dunnett’s t-test.

Values shown are the mean ± standard error of mean (SEM).

*, p ≤ 0.05

****, p ≤ 0.0001.

### Exogenous galectin-3 effects on cell viability

**Fig 4:**
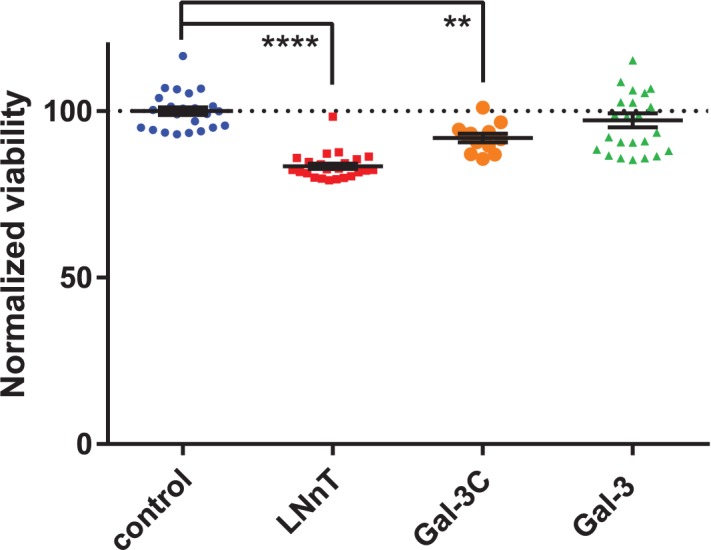
Viability of cells treated with LNnT, Gal-3C, and Gal-3. Cells were treated for 21 h with buffer control, Gal-3 (50 μg mL^-1^), Gal-3C (50 μg mL^-1^), and LNnT (100 mM). Viability of each condition were measured using MTS assay.[88] Viability for each condition was normalized and compared to buffer control; *, p ≤ 0.05; **, p ≤ 0.01; ***, p ≤ 0.005; ****, p ≤ 0.0001.

To further understand the differential regulation of Gal-3 and Gal-3C, we investigated the viability of cells after addition of exogenous Gal-3, Gal-3C, and LNnT using identical conditions to the migration assays. We observed a significant decrease in viability of cells treated with LNnT and Gal-3C (**Table 3, Fig 4**). Importantly, addition of Gal-3 did not change the viability of cells over the course of incubation. The toxicity of LNnT is most likely due to osmotic stress due to the high concentration of the oligosaccharide (100 mM). Control experiments with similar concentrations of sucrose and lactose also showed toxicity in this assay (**Table D in S1 File**). Both Gal-3C and LNnT decreased cell viability, albeit to different degrees; and this finding suggested that the decreased migration of cells in these two conditions is, in part, due to reduced cell viability.

**Table 3:** Viability of cells under cell migration conditions.

**Condition**	**N**	**viability**†
**Buffer control**	24	100 ± 3
**LNnT**	24	84 ± 1****
**Gal-3C**	12	92 ± 1**
**Gal-3**	24	98 ± 2

†Samples were normalized and compared to the indicated control for significance using a Dunnett’s t-test.

Values shown are the mean ± standard error of mean (SEM).

**, p ≤ 0.01

****, p ≤ 0.0001.

## Discussion

**Fig 5:**
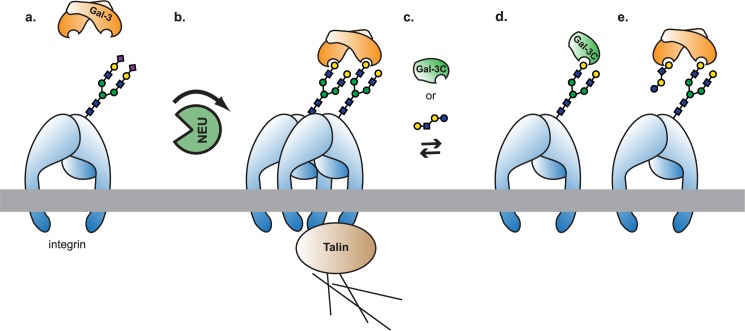
Model of Gal-3 interactions with integrin. (**a.**) Glycosylated receptors, such as the integrins, will have reduced binding sites for Gal-3 if they are heavily sialylated. (**b.**) Removal of sialic acids by neuraminidase enzymes (or decreased SiaT activity) will increase the number of Gal-3 binding sites present, and should increase oligomerization (only a dimer is shown for clarity). Oligomers likely interact with cytoskeletal regulators, including talin,[89] which lead to increased mobility through active processes. (**c.**) Addition of exogenous Gal-3C or a competitive binder (e.g. LNnT) will disrupt the formation of oligomers. This will occur either by (**d.**) competition for dimerization sites or (**e.**) blocking dimer binding sites.

Using measurements of β1-integrin lateral mobility, we have found that galectins alter the diffusivity of integrin receptors in the membrane. In HeLa cells, the α5β1 integrin showed a decrease in lateral mobility when exogenous Gal-3C was added. While the truncated Gal-3C reduced mobility, the full-length Gal-3 protein (with both the CRD and N-terminal domain) enhanced integrin mobility. Treatment of cells with high affinity oligosaccharides for Gal-3 increased integrin lateral mobility, although this effect may be complicated by toxicity or the presence of other ligands. Addition of a bacterial neuraminidase enzyme, NanI, resulted in decreased high-mobility integrins, but no change in the mean diffusion coefficient. Quantification of integrin cluster size on cells treated with Gal-3 found increased clustering, while Gal-3C alone and LNnT had no apparent effects. Thus, one conclusion from this work is that Gal-3 was able to increase integrin clustering through increased diffusivity of the receptor. We explored the functional consequences of these changes to adhesion using a FN–β1-integrin cell migration assay. We observed that Gal-3 treatment increased β1-integrin mediated cell migration, while Gal-3C and LNnT inhibited migration. Inhibition of cell migration by Gal-3C may be the result of blocking native Gal-3 clustering of the adhesion receptor (**Fig 5**). Treatment with LNnT, a high affinity ligand for Gal-3, may also reduce integrin mobility through blocking of Gal-3–integrin interactions. Alternative mechanisms involving glycolipids may also be possible.[68] Together, these results provide quantitation of the effect of Gal-3 on integrin lateral mobility and organization in the membrane; and reveal that Gal-3 likely triggers an active process which results in increasing lateral mobility of integrins.

Earlier work from our group has found that the lateral mobility of the α5β1 integrin was reduced by the activity of a native neuraminidase, NEU3.[44] This finding was ascribed to changes in glycolipid composition which resulted in altered cytoskeletal interactions and endocytosis of the integrin.[68] To see if changes to integrin glycosylation were occurring here, we confirmed that NanI and NEU3 treatment altered glycosylation of the β1 chain of the integrin receptor (Figure C in S1 File).[44] NanI treatment resulted in a change to the diffusion profile of the integrin receptor, but did not cause a significant decrease in average mobility. We speculate that NanI treatment resulted in the exposure of new cryptic Gal-3 binding sites on receptors besides the integrin, which could result in sequestration of the native Gal-3 away from integrin clusters. Our integrin trajectories suggest substantial confinement, consistent with cytoskeletal interactions. Previous models of Gal-3–integrin interactions have proposed that clustering of the integrin activates downstream cytoskeletal components (e.g. FAK, Rac-1),[52] and may involve interactions with glycosphingolipids.[21] Thus, it is likely that the increased mobility of integrin clusters was due to changes in cytoskeletal processes linked to endocytosis.[40, 69]

Galectin-3 has previously been found to interact directly with the β1 integrin (CD29).[39, 70] CD7 is also known to be a co-receptor and ligand for Gal-3,[40, 70] but does not appear to be a ligand for Gal-3 on T cells.[39] Co-localization of the β1 integrin with Gal-3 has been observed by fluorescence microscopy,[52] but the effect of Gal-3 on the lateral mobility of β1 integrin has not been investigated. Our results indicate that exogenous forms of Gal-3 (both the holoprotein and the CRD) were capable of altering integrin mobility. The full-length Gal-3 and truncated Gal-3C had opposite effects on lateral mobility and integrin-mediated cell migration. Gal-3 treatment resulted in increased cell migration, whereas Gal-3C was inhibitory (**Fig 4**). These findings are consistent with previous work that found Gal-3 can promote lamellipodia formation[52] and cell migration.[71] Furthermore, Gal-3C is known to inhibit cell migration and cell growth, and shows mild toxicity above 25 μg mL^-1^.[72]

Native expression of galectins likely contributes to integrin clustering and mobility. Our proteomics data confirmed endogenous expression of Gal-1 and Gal-3, suggesting that untreated cells have a native Gal-3–integrin lattice which may be disrupted through the addition of ligands such as LNnT. The prevalence of the galectin lattice will be regulated by the expression of the lectin and the number of binding sites on cellular receptors. The native Gal-3 binding sites should be regulated by the activity of MGAT5,[52, 73] sialyltransferase, and neuraminidase enzymes.[8] Treatment of cells with exogenous NanI should have increased the number of Gal-3 binding sites. Furthermore, native neuraminidase enzymes which directly modify the integrin, or other adhesion receptors, may influence adhesion through this mechanism.[74, 75]

We used two methods to quantitate changes to the Gal-3–integrin lattice on cells: SPT and fluorescence microscopy. Single-particle tracking provides a profile of different diffusive behaviors occurring on the cell surface.[56, 57, 76] For integrin receptors, this can often manifest as a heterogeneous population which may have distinct sub-populations present.[77, 78] In this study, we found that integrin diffusion could be analyzed as a single log-normal distribution of diffusion coefficients. This finding is consistent with our previous work on β1 integrins, and may be due to the shorter timescale of the measurement used here.[44] Lateral mobility measurements show significant changes in the presence of Gal-3, likely due to lattice formation and cytoskeletal regulators. Our analysis of Gal-3C treatment by fluorescence microscopy found no substantial changes to integrin clustering unless cytoD was co-administered. Due to the optical resolution of microscopy, these changes in clustering are biased for large oligomers.

The data presented here provide quantitative support for the formation of a Gal-3–integrin lattice on the surface of live cells. Our data suggest that disruption of a native galectin–integrin lattice may occur in the presence of competitive oligosaccharide ligands. The LNnT oligosaccharide is found in human milk,[79] and these data may lend support to the role of galectins as HMO receptors.[80] Most importantly, we found that exogenous Gal-3, was able to increase α5β1 integrin clustering and lateral mobility. The effects of Gal-3 on integrin organization also induce changes in integrin activity, as detected by cell migration. Exogenous Gal-3 increased cell migration, while the truncated Gal-3C inhibited cell migration. Our findings suggest that aggregation and disruption of the galectin–integrin lattice by high affinity ligands or competitive inhibitors could be used to disrupt cell migration. Future work should address the effects of Gal-3 on additional co-receptors to determine if the lattice manifests target-specific effects on lateral mobility, and could explore the activity of higher affinity ligands for Gal-3.[81]

## Materials & methods

### Reagents and cell lines

Phorbol 12-myristate 13-acetate (PMA; Sigma-Aldrich, Oakville, Ontario, Canada) and *Clostridium perfringens* neuraminidase (pfNeu, the NanI isoform;[44] Sigma-Aldrich, Oakville, Ontario, Canada) were dissolved in PBS as stock solutions. Lactose (Gal-β1,4Glc; Sigma-Aldrigh, Oakville, Ontario, Canada) and lacto-N-neotetraose (LNnT; Gal-β1,4GlcNAc-β1,3Gal-β1,4-Glc; Elicityl, Grenoble, France) were both used at a final concentration of 100 mM. Cytochalasin D (cytoD; Sigma-Aldrich, Oakville, Ontario, Canada) was dissolved in a dimethyl sulfoxide (DMSO) stock solution and used after dilution to a final concentration of 500 ng mL^-1^ with 0.05% DMSO in cell media or buffer.

HeLa cells were a kind gift of Prof. R.E Campbell (University of Alberta). HeLa cells were cultured and maintained in Dulbecco's modified Eagles medium (DMEM; Gibco, Invitrogen, USA) containing penicillin/streptomycin (Gibco, Invitrogen, USA) and 10% fetal bovine serum (Hyclone, Thermo, USA). Cells were used between 3 and 7 passages, and grown at 37°C in a humidified incubator with 5% CO_2_.

Recombinant human Galectin 3 C-terminal domain (Gal-3C), and Galectin 3 wild type (Gal-3) were produced as previously described with minor modifications (see S1 File).[63, 82]

### Proteomics analysis of Gal expression in HeLa

Carbohydrate modified sepharose gel was prepared as previously reported.[83] One mL of settled Sepharose CL-6B (GE Healthcare Life Sciences, Piscataway, N.J.) was thoroughly washed with water in a sintered funnel and then re-suspended in 0.5 M carbonate buffer (pH 11) with 100 μL of divinyl sulfone (Sigma Aldrich, Milwaukee, WI). The mixture was agitated for 70 min, after which the resin was transferred to a sintered funnel and extensively washed with water. The moist cake was suspended in a 1 mL solution of the indicated carbohydrate (1.11 mmol mL^-1^ in 0.5 M carbonate buffer, pH 10) and left agitating for 18 h. The resin was washed again with distilled water over a sintered funnel, and the moist cake re-suspended in carbonate buffer (1 mL, 0.5 M, pH 8.5) with 2-mercaptoethanol (6 μL). After 2 hrs the sample was washed with distilled water and stored in 20% ethanol solution.

Affinity chromatography was performed by equilibrating the resin with running buffer (20 mL; 0.5 M NaCl, 1 mM CaCl_2_, 20 mM Tris-HCl, pH 8), followed by injection of the protein solution (5 mL). The column was washed with additional running buffer (10 mL), and then eluted with elution buffer (20 mL; 0.2 M Lac, 3M NaCl, 50 mM Tris-HCl, pH 8) followed by glycine buffer (20 mL; 0.1 M glycine, pH 2.3). The flow-through from the elution buffer was collected and concentrated by ultrafiltration.

In-solution digest of the eluent was performed according to reported protocols.[84] Briefly, the protein solution was precipitated with a chloroform and methanol mixture. The protein pellet was dried over a stream of nitrogen and then dissolved in 8 M urea, followed by addition of iodoacetamide (20 mM final concentration) and DTT (10 mM final concentration). The resulting protein mixture was digested with trypsin and then quenched with formic acid (10% v/v) and used for analysis by ESI-MS. Data was analyzed with MASCOT.[85]

### Cell treatment and single particle tracking

Cell samples were prepared by washing 1 x 10^5^ cells into fresh media after centrifugation at 1200 rpm for 2 min, which were then allowed to settle onto a poly-L-lysine-treated (10 μg mL^-1^) confocal dish overnight at 37°C. Cells were treated by incubation at 37°C for 0.5 or 2 hours as indicated. For oligosaccharide incubations, cells were re-suspended in 1 mL of PBS, or oligosaccharides (100 mM; Lac, 34.23 mg mL^-1^; LNnT, 70.76 mg mL^-1^) in PBS. For Gal-3 and Gal-3C incubations, cells were resuspended in 1 mL of PBS, or Gal-3 (25 μg mL^-1^ or 50 μg mL^-1^) in PBS, or Gal-3C (50 μg mL^-1^) in PBS. After incubation, all treated cells were washed 3 times with fresh PBS before labelling and analysis.

Cells were labeled with Cy5-anti-CD49e (clone: SAM-1; 100 ng mL^-1^) to stain for the α4 integrin complex for 30 minutes at room temperature in the dark. Attempts to label the cells with the Cy5-F(ab) were unsuccessful, so the intact IgG was used for all experiments. The Cy5-antibody conjugate was generated using an NHS ester of Cy5 (GE Healthcare, Buckinghamshire, UK) following the manufacturer’s protocol. Cells were washed 3 times with PBS buffer after labelling, and then observed on a Nikon ECLIPSE Ti microscope system by total internal reflection fluorescence (TIRF) at 37°C, with a 60x oil objective at 633 nm. Video data were processed using and NIS-Elements v3.5 (Nikon, USA) for 10 sec and analyzed with u-track[76] with custom scripts written in MATlab (2012b).[78, 86] Trajectories shorter than 20 steps were excluded. The intensity of the trajectories was used to exclude the top and bottom 5% of trajectories from the analysis.

### Immunofluorescence imaging

Immunofluorescence imaging for cluster analysis was performed using an identical protocol as SPT with 100 ng mL^-1^ of the antibody conjugate. Random fields were selected to obtain images of approximately ten cells for analysis. Cells were selected based on DIC and fluorescence staining. Images of individual cells were processed in ImageJ by applying a threshold and processing using the analyze particle function to measure clusters larger than 4 pixel^2^ (0.7 μm^2^). Data were analyzed as the average cluster size (μm^2^ cluster^-1^) on a per cell basis (n = 5–15 cells).

### Cell migration studies

Migration studies were done using an Oris 96-well plate assay kit (Platypus Technologies, USA) using the manufacturers protocol. Briefly, the migration surfaces were coated with fibronectin (10 μg mL^-1^ in PBS, Calbiochem, USA) for 2 h and 100 μL of IgG-free BSA (200 μg mL^-1^ in PBS, Sigma Aldrich, USA) for 1 h. After that, stoppers were placed in each well, and plates were incubated with 50 x 10^3^ cells mL^-1^ for 18 h. Stoppers were then removed and images of each well were taken under bright field using a 4x objective with a Nikon T1 Eclipse inverted microscope as time zero. The experimental plate was incubated for 21 h to allow cells to migrate. Cells were treated with buffer alone or with Gal-3 (50 μg mL^-1^), Gal-3C (50 μg mL^-1^), and LNnT (100 mM) in DMEM supplemented with 10% heat inactivated FBS. Images of plate wells were imaged again and compared with the images at time zero. The difference in area was measured using Image J software. For each condition the experiment was conducted in at least triplicate measurements performed on separate days to account for intra-day and inter-day variabilities. Migration area for each replicate was normalized to that of an intra-day buffer control. Normalized replicates were then pooled together for statistical analysis.

Normalization of data was performed by using the following equation: $r=\left(\frac{A_{0}-A_{21}}{M_{B}}\right)\times 100$. Where, r is the normalized migration, A_0_ is the cell-free area at time zero (μm^2^), A_21_ is the cell-free area at 21 h incubation (μm^2^), and M_B_ is the mean cell-free area in the control after 21 h (μm^2^).

### Viability studies

Toxicity of compounds was assayed using identical conditions as those in migration studies. Viability of cells was determined using a CellTiter 96 AQ_ueous_ Cell Proliferation Assay kit (MTS) from (Promega, USA) using the manufacturers protocol. Briefly, wells of a 96-well plate were seeded with 100 μL of 50 x 10^4^ cells mL^-1^, and incubated for 18 h in a 5% CO_2_ incubator at 37°C. Cells were then treated with Gal-3 (50 μg mL^-1^), Gal-3C (50 μg mL^-1^), and LNnT (100 mM) in DMEM supplemented with 10% heat inactivated FBS for 21 h. After incubation for 21 h, 20 μL of an MTS solution was added to each well and incubated for 2 h. The absorbance of soluble formazan produced by viable cells from MTS was measured at 490 nm using a SpectraMax M2 (Molecular Devices) plate reader.

For each condition the experiment was conducted in at least triplicate measurements on multiple days to account for intra-day and inter-day variabilities. Absorbance for each replicate was normalized to that of the intra-day buffer control. All replicates of a condition were then pooled together for statistical analysis. The data were normalized by dividing the absorbance of the sample by the mean absorbance of the buffer control.

## Supporting information

S1 File The file includes proteomics data (Table A), analysis of fluorescence microscopy data (Table B), lateral mobility controls (Table C), and cytotoxicity controls (Table D), fluorescence microscopy (Figure A), lateral mobility data with NanI treatment (Figure B), and western blots of α5β1 integrin after NEU treatment (Figure C).
(PDF)

S2 File Diffusion data. A table of fit microdiffusion values for the conditions shown in Fig 1.
(XLSX)
