# Supplementary material for: Galectin-3 alters the lateral mobility and clustering of β1-integrin receptors
Source: PLoS One. 2017 Oct 10;12(10):e0184378. doi: 10.1371/journal.pone.0184378 (PMC5634555; doi:10.1371/journal.pone.0184378)
Supplement: S1 File — The file includes proteomics data (Table A), analysis of fluorescence microscopy data (Table B), lateral mobility controls (Table C), and cytotoxicity controls (Table D), fluorescence microscopy (Figure A), lateral mobility data with NanI treatment (Figure B), and western blots of α5β1 integrin after NEU treatment (Figure C). (PDF) [file pone.0184378.s001.pdf]

Supporting information for:

Galectin-3 alters the lateral mobility and clustering of β1-integrin receptors

Esther H. Yang, Julia Rode†, Md. Amran Howlader, Marina Eckermann[¶], Jobette T. Santos‡, Daniel Hernandez Armada, Ruixiang Zheng, Chunxia Zou, and Christopher W. Cairo*

Contents

Preparation of Gal-3 and Gal-3C proteins 2

Table A: Shotgun proteomics of HeLa cell lysate 3

Table B: Cluster Analysis of integrins by TIRF 4

Table C: Lateral mobility osmotic controls 4

Table D: Cytotoxicity osmotic controls 4

Fig A: Galectin-3C induces clustering of integrin receptors on cells. 5

Fig B: Lateral mobility of integrins with NanI and Gal-3C treatments 6

Fig C: Changes in glycosylation to α5β1 integrin with NEU treatment 7

References 8

Preparation of Gal-3 and Gal-3C proteins

The genes of the full length human galectin-3 (Gal-3) or the C-terminal fragment (Gal-3C, residues 107-250) were optimized and synthesized by Genscript Inc. and then subcloned into a *p*ET30b vector. The Gal-3C protein was overproduced in *E.coli* BL21 (DE3), following previous reports.[1] A single colony was used for inoculation of an overnight culture in LB medium. Bacterial growth was monitored by absorbance at 600 nm (A_{600}). At A_{600} ~ 0.6, the culture was induced by adding IPTG to a final concentration if 1 mM, followed by culture overnight, and centrifugation 20 °C. The cell pellets were resuspended in MEPBS (1 × PBS, pH 7.2, 2 mM β-mercaptoethanol, 4 mM EDTA) and lysed using a cell disruptor. The lysate was centrifuged at 100,000 x g, and the supernatant was loaded onto an equilibrated lactosyl-Sepharose column.[2] The column was washed with MEPBS, and eluted with MEPBS containing 200 mM lactose. The eluant was dialyzed into PBS buffer before experiments, or stored at -80 °C. The nucleotide sequences for each protein are given below.

Gal-3 nucleotide sequence

```
ATGGCAGATAACTTCTCGCTGCATGACGCACTGTCGGGCTCGGGTAATCCGAATCCGCAGGGCTG
GCCGGGCGCTTGGGGTAATCAACCGGCAGGTGCCGGCGGTTATCCGGGTGCTTCTTATCCGGGCG
CATACCCGGGTCAGGCTCCGCCGGGTGCATACCCGGGTCAAGCACCGCCGGGTGCATATCCGGGT
GCACCGGGTGCTTACCCGGGTGCACCGGCTCCGGGTGTGTATCCGGGTCCGCCGTCAGGCCCGGG
TGCCTACCCGAGCTCTGGTCAGCCGTCGGCAACCGGTGCATATCCGGCAACGGGTCCGTACGGTG
CACCGGCAGGTCCGCTGATTGTTCCGTATAACCTGCCGCTGCCGGGCGGTGTGGTTCCGCGTATG
CTGATTACCATCCTGGGCACGGTCAAACCGAACGCTAATCGTATTGCGCTGGATTTTCAACGCGG
TAACGACGTGGCGTTTCATTTCAACCCGCGCTTCAATGAAAACAATCGTCGCGTCATCGTGTGCA
ATACCAAACTGGATAACAATTGGGGCCGTGAAGAACGCCAGAGTGTTTTTCCGTTCGAATCCGGT
AAACCGTTTAAAATCCAAGTTCTGGTCGAACCGGATCACTTCAAAGTGGCCGTTAATGACGCACA
TCTGCTGCAGTATAACCACCGTGTCAAAAAACTGAATGAAATTAGTAAACTGGGCATTTCTGGCG
ACATTGACCTGACCTCGGCGTCCTACACGATGATTTAATGA
```

Gal-3C nucleotide sequence

```
ATGTATGGCGCTCCGGCTGGTCCGCTGATTGTTCCGTACAACCTGCCGCTGCCGGGTGGCGTCGT
CCCGCGTATGCTGATTACCATCCTGGGCACCGTGAAACCGAACGCGAATCGTATTGCCCTGGATT
TTCAGCGCGGTAACGACGTTGCCTTTCATTTCAACCCGCGTTTCAATGAAAACAATCGTCGCGTG
ATCGTTTGCAATACGAAACTGGATAACAATTGGGGCCGTGAAGAACGCCAGAGCGTCTTTCCGTT
CGAATCTGGTAAACCGTTTAAAATTCAAGTCCTGGTGGAACCGGATCACTTCAAAGTTGCAGTCA
ACGACGCTCATCTGCTGCAATATAACCACCGCGTTAAAAAACTGAATGAAATCAGTAAACTGGGC
ATTAGCGGTGACATTGACCTGACCTCGGCGTCCTACACGATGATTTGATGA
```

Gal-3 protein sequence
MADNFSLHDALSGSGNPNPQGWPGAWGNQPAGAGGYPGASYPGAYPGQAPPGAYPGQAP
PGAYPGAPGAYPGAPAPGVYPGPPSGPGAYPSSGQPSATGAYPATGPYGAPAGPLIVPY
NLPLPGGVVPRMLITILGTVKPNANRIALDFQRGNDVAFHFNPRFNENNRRVIVCNTKL
DNNWGREERQSVFPFESGKPFKIQVLVEPDHFKVAVNDAHLLQYNHRVKKLNEISKLGI
SGDIDLTSASYTMI

Gal-3C protein sequence
MYGAPAGPLIVPYNLPLPGGVVPRMLITILGTVKPNANRIALDFQRGNDVAFHFNPRFN
ENNRRVIVCNTKLDNNWGREERQSVFPFESGKPFKIQVLVEPDHFKVAVNDAHLLQYNH
RVKKLNEISKLGISGDIDLTSASYTMI

Table A: Shotgun proteomics of HeLa cell lysate

Protein accession	***LNnT†***	***Lac†***	***Seph†***
ANXA2_HUMAN	0	35	0
H12_HUMAN	360	0	0
H13_HUMAN	386	0	0
H14_HUMAN	372	221	0
H15_HUMAN	411	203	0
H1T_HUMAN	144	0	0
H2B1C_HUMAN	29	42	0
LEG1_HUMAN	22	195	0
LEG3_HUMAN	0	190	0
RL23A_HUMAN	0	133	0
TCP4_HUMAN	0	67	0
YBOX1_HUMAN	86	46	0

† Mascot protein scores are shown for three separate experiments using affinity columns. Columns were either unmodified sepharose (Seph), Lac, or LNnT columns. See Materials and Methods for details.

Table B: Cluster Analysis of integrins by TIRF

Condition	n	mean‡	p†
**control**	12	0.42 ± 0.02	-
**Gal-3C**	11	0.65 ± 0.05***	0.0002
**Gal-3C + CytoD**	9	0.48 ± 0.09	0.52
**control (PBS)**	15	0.33 ± 0.03	-
**Lac**	5	0.36 ± 0.03	0.50
**LNnT**	7	0.37 ± 0.02	0.30
**CytoD**	10	0.48 ± 0.05**	0.0057
**Gal-3+CytoD**	9	0.45 ± 0.02**	0.0034
**Gal-3**	7	0.6 ± 0.1*	0.0122

‡Mean area of clusters per cell ± SEM, [μm^2]. n represents the number of individual cells.

†Data were compared to the appropriate control using a t-test to determine p values; *, $p \leq 0.05$; **, $p \leq 0.01$; ***, $p \leq 0.005$; ****, $p \leq 0.0001$. Scale bar = 5 μm.

Table C: Lateral mobility osmotic controls

Condition	n	mean‡	log mean‡
**control (PBS)**	174	1.9 ± 0.3	0.8 ± 0.06
**Lac**	213	2.2 ± 0.6	0.6 ± 0.04
**LNnT**	135	3.7 ± 0.5***	1.3 ± 0.1***

‡, Units are [$x\ 10^{-10}\ cm^2\ sec^{-1}$] or [$x\ 10^{-2}\ \mu m^2\ sec^{-1}$].

*, p values were calculated by comparison of two normal populations of raw or log-transformed data as indicated. Samples were compared to the indicated control for significance using a student's t-test. *, $p \leq 0.05$; **, $p \leq 0.01$; ***, $p \leq 0.005$; ****, $p \leq 0.0001$.

†, Median values were calculated as described in supporting information.

Table D: Cytotoxicity osmotic controls

Condition	n	mean
**control (PBS)**	32	100 ± 14
**Lac 100 mM**	32	61 ± 12****
**Sucrose 100 mM**	32	65 ± 9****

†Data were compared to the appropriate control using a t-test to determine p values; *, $p \leq 0.05$; **, $p \leq 0.01$; ***, $p \leq 0.005$; ****, $p \leq 0.0001$. Scale bar = 5 μm.

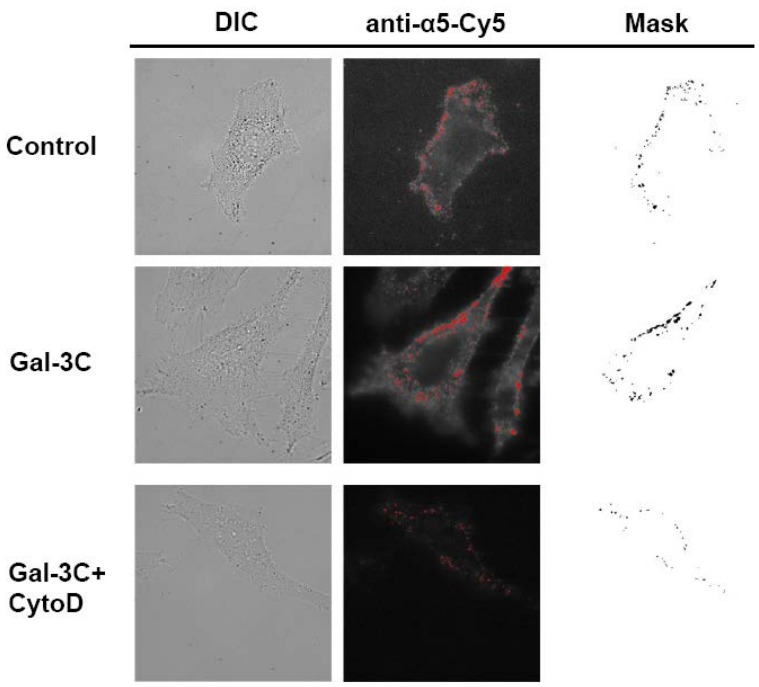

Fig A: Galectin-3C induces clustering of integrin receptors on cells.

Cells were stained using the same antia5-Cy5 conjugate employed for tracking experiments. Ten fields of stained cells were analyzed using ImageJ to identify clusters and measure their size. Sample images from each treatment are shown, and quantification of these images is shown in Fig 2. Galectin-3C resulted in an increase in the size of integrin clusters.

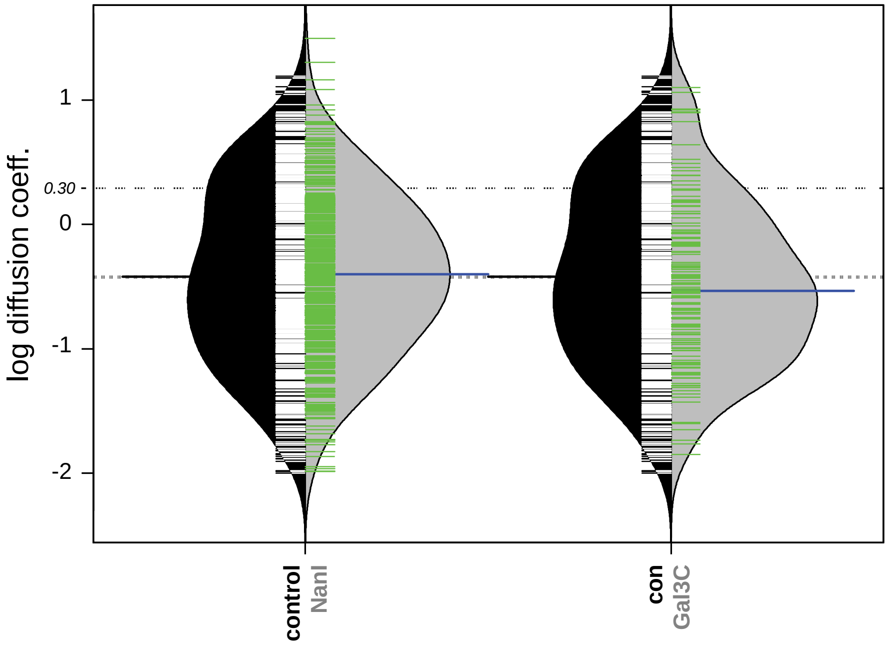

Fig B: Lateral mobility of integrins with NanI and Gal-3C treatments

The lateral mobility of integrins were determined using SPT, and the data from Table 1 are shown using a beanplot. Each sample population (grey) is shown compared to the control (black). The logarithmic median of the diffusion coefficients is indicated by a solid line for each population.[3] Each population is shown with a density estimate and horizontal lines indicate individual diffusion coefficient measurements. Diffusion coefficients are given as log(D), where D is in units of x 10^{-10} [cm^2s^{-1}] or x 10^{-2} [μm^2s^{-1}]. There is a notable decrease in the number of trajectories observed >2 x 10^{-10} [cm^2s^{-1}] in both conditions (indicated by the dashed line at 0.30).

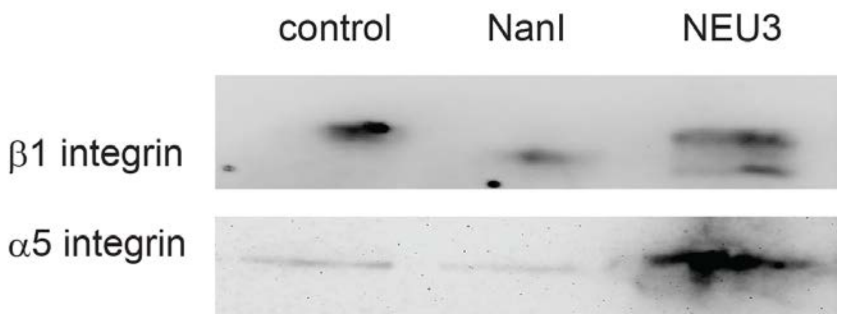

Fig C: Changes in glycosylation to α5β1 integrin with NEU treatment

To detect changes in the molecular weight of the α5β1 integrin receptor, we performed western blots of the protein isolated from HeLa cells. Briefly, cells were treated with the indicated enzyme condition for 3 h at 37 °C. Cells were harvested and lysed with lysis buffer (50 mM Tris, pH 8.0, 150 mM NaCl, 1% Triton X-100, 0.5% sodium deoxycholate, 0.1% SDS and a protease inhibitor cocktail). The lysate samples were concentrated and then separated by SDS-PAGE, and transferred to a nitrocellulose membrane. The membranes were probed with (top) anti-CD29 (β1 integrin chain; clone EP1041Y, Abcam), or (bottom) anti-CD49d (α5 integrin chain; clone EPR7854, Abcam), and a goat anti-rabbit HRP secondary antibody (clone ab6721, Abcam). The blot was developed using ECL (Bio-Rad, USA). The blot is shown with the following conditions: lane 1, control; lane 2, NanI treatment (pH 4.5); lane 3: Neu3 (pH 4.5). Both lanes 2 and 3 show the appearance of a new, lower molecular weight, band for the β1 integrin consistent with desialylation of the integrin.[4] The α5 chain did not show any apparent changes in molecular weight.

References

(1) Diehl, C., Genheden, S., Modig, K., Ryde, U., and Akke, M. (2009) Conformational entropy changes upon lactose binding to the carbohydrate recognition domain of galectin-3. *J. Biomol. NMR 45*, 157-169.

(2) Hernandez Armada, D., Santos, J. T., Richards, M. R., and Cairo, C. W. (2015) Protecting group-free immobilization of glycans for affinity chromatography using glycosylsulfonohydrazide donors. *Carbohydr. Res. 417*, 109-116.

(3) Kampstra, P. (2008) Beanplot: A boxplot alternative for visual comparison of distributions. *Journal of Statistical Software 28*, 1-9.

(4) Jia, F., Howlader, M. A., and Cairo, C. W. (2016) Integrin-mediated cell migration is blocked by inhibitors of human neuraminidase. *Biochimica et Biophysica Acta (BBA) - Molecular and Cell Biology of Lipids 1861*, 1170-1179.
